# A Novel Hydro-Thermal Synthesis of Nano-Structured Molybdenum-Iron Intermetallic Alloys at Relatively Low Temperatures

**DOI:** 10.3390/ma16072736

**Published:** 2023-03-29

**Authors:** A. A. El-Geassy, K. S. Abdel Halim, Abdulaziz S. Alghamdi

**Affiliations:** 1 Central Metallurgical Research and Development Institute (CMRDI), P.O. Box 87, Helwan 11421, Egypt; elgeassy@hotmail.com; 2 College of Engineering, University of Ha’il, P.O. Box 2440, Hail 55476, Saudi Arabia; a.alghamdi@uoh.edu.sa

**Keywords:** iron, molybdenum, reduction, nanostructured materials, thermal powder technology, intermetallics, alloys and compounds, kinetics, mechanism, materials characterization, gas–solid reactions

## Abstract

Nano-structured Mo/Fe intermetallics were synthesized from precursors that contained 72/28% and 30/70% molar ratios of Mo/Fe, which were given as precursors A and B, respectively. These precursors were prepared from the co-precipitation of aqueous hot solutions of ammonium heptamolybdate tetrahydrate (AHM) and ferrous oxalate. The dry precipitates were thermally treated using TG-DSC to follow up their behavior during roasting, in an Ar atmosphere of up to 700 °C (10° K/min). The TG profile showed that 32.5% and 55.5% weight losses were measured from the thermal treatment of precursors A and B, respectively. The DSC heat flow profile showed the presence of endothermic peaks at 196.9 and 392.5–400 °C during the thermal decomposition of the AHM and ferrous oxalate, respectively. The exothermic peak that was detected at 427.5 °C was due to the production of nano-sized iron molybdate [Fe_2_(MoO_4_)_3_]. An XRD phase analysis indicated that iron molybdate was the only phase that was identified in precursor A, while iron molybdate and Fe_2_O_3_ were produced in precursor B. Compacts were made from the pressing of the nano-sized precursors, which were roasted at 500 °C for 3 h. The roasted compacts were isothermally reduced in H_2_ at 600–850 °C using microbalance, and the O_2_ weight loss that resulted from the reduction reactions was continuously recorded as a function of time. The influence of the reduction temperature and precursor composition on the reduction behavior of the precursors was studied and discussed. The partially and completely reduced compacts were examined with X-ray powder diffraction (XRD), a reflected light microscope (RLM), and a scanning electron microscope (SEM-EDS). Depending on the precursor composition, the reduction reactions of the [Fe_2_(MoO_4_)_3_] and Fe_2_O_3_ proceeded through the formation of intermediate lower oxides, prior to the production of the MO/Fe intermetallic alloys. Based on the intermediate phases that were identified and characterized at the early, intermediate, and final reduction degrees, chemical reaction equations were given to follow up the formation of the MoFe and MoFe_3_ intermetallic alloys. The mechanism of the reduction reactions was predicted from the apparent activation energy values *(Ea*) that were computed at the different reduction degrees. Moreover, mathematical formulations that were derived from the gas–solid reaction model were applied to confirm the reduction mechanisms, which were greatly dependent on the precursor composition and reduction temperature. However, it can be reported that nano-structured MoFe and MoFe_3_ intermetallic alloys can be successfully fabricated via a gas–solid reaction technique at lower temperatures.

## 1. Introduction

Recently, the fabrication of metallic alloy materials has been considered to be an important issue for both scientific and industrial applications [1,2,3,4,5]. A gas–solid reaction technique is considered to be a promising process and an environmentally friendly approach for the manufacturing of binary and tertiary metal alloys, as well as for the production of nano-structured intermetallics [6,7,8,9,10,11]. This technique is also widely applied to the production of metals from metal oxides and their ores [12,13]. The produced alloys have enough active centers to achieve good chemical bonding between the dispersed material and the matrix, and are widely applied within many engineering industries [14]. The composition and the morphology of the produced intermetallics can be controlled by adjusting the reaction parameters, such as the molar ratio of the reactants, the particle size, the reaction temperature, and the gas composition [15,16,17]. The produced tailor-made alloys can be further heat treated to improve their mechanical properties, prior to the manufacturing of micro-structured tools [18,19]. However, great attention has recently been paid to the use of these intermetallic alloys in semiconductors, photo-voltaic cells, special ceramics, and other chemical industries [20,21]. Intermetallic alloys have unique properties, particularly at high temperatures; hence, they can be developed as bases in the manufacturing of advanced materials that have different applications within the energy sector (power generation), transportation sector (automobile and aircraft production), and other engineering industries [22].

The pre-determined compositions of certain alloys can be produced from precursors that contain different molar ratios of intermetallics, via chemical co-precipitation, or sol–gel, thermal, and/or mechanical routes [23,24]. The co-precipitation technique is one of the most promising processes, due to its low cost, suitability for mass production, and production of high-purity alloys. About 80% of commercial molybdenum is used in metallurgical industries (35% in structural steel, 25% stainless steel, 9% in tools and high-speed steels, 6% in cast iron, and 5% in superalloys) [25]. Molybdenum is also used in the production of steel alloys, due to its high corrosion resistance and weldability, which increase the lattice strain and consequently increase the energy that is required to dissolve the iron atoms from the surface. On the other hand, molybdenum-based alloys that do not contain iron have limited applications. Morales [26] studied the production of an Fe_2_Mo alloy from a reduction of Fe_2_MoO_4_ with H_2_ gas in a fluidized bed reactor. FeMo intermetallics with different Fe/Mo molar ratios were synthesized from the reduction of the FeO-Fe_2_MoO_4_-MoO_2_ mixtures with H_2_, at 600–750 °C. It was reported that the gas–solid reaction route, in combination with a powder technique, is a promising process towards the production of novel metallic alloys, such as the Fe_2_Mo intermetallic with micro- and nanocrystalline grains. In general, binary and tertiary ferrous and non-ferrous alloys can be produced from oxide mixtures via different techniques, such as mechanical alloying and/or electrodeposition techniques [27,28].

Nowadays, the gas–solid reduction technique is widely applied in ironmaking processes, in order to produce directly reduced iron (DRI) or so-called sponge iron for special steel production [29,30,31,32,33]. In this process, iron oxide (Fe_2_O_3_) is directly reduced with H_2_ and/or CO to metallic iron, via intermediate reaction products (magnetite and/or wűstite) at relatively low temperatures.

The present study aims at a synthesis of the MoFe and MoFe_3_ intermetallic alloys from the isothermal reduction of precursors that contain different molar ratios, with H_2_ at relatively low temperatures. The reduction reaction mechanisms of the precursors were elucidated from the correlation between the apparent activation energy values (E_a_) and the application of the gas–solid heterogeneous mathematical formulations, which were derived from the gas–solid reaction model.

## 2. Materials and Experimental Procedure

### 2.1. Materials and Characterizations

Ammonium heptamolybdate tetrahydrate (AHM), with the chemical formula [(NH_4_)_6_Mo_7_O_24_·4H_2_O], and ferrous oxalate dihydrate (FeC_2_O_4_·2H_2_O) (Aldrich, Munich, Germany, >99.5% purity) were used for the preparation of two precursors containing 72/28% and 30/70% Mo/Fe molar ratios, named as precursor A and precursor B, respectively. The pre-determined weights of the AHM and ferrous oxalate were dissolved in hot, bi-distilled water. The aqueous mixture was heated up to 70 °C with continuous stirring until it reached complete dryness. The different phases in the dry precipitate were identified by XRD (XRD, PAN Analytical Empyrean, Eindhoven, The Netherlands, 40 kV, 40 mA, Cu target, 1.543 A). A TG-DSC simultaneous thermal analysis (STA 504 BÄHR Thermo-analyze, GmbH, Hüllhorst, Germany) was used to follow up the thermal behavior of the precursors during heating, in an Argon atmosphere of up to 700 °C. In this technique, the sample and inert sample (Al_2_O_3_) were subjected to a defined temperature program (isothermal or dynamic), and the changes in the mass and the heat turnover from the physical conversion and chemical reaction were recorded at the given times. A reflected light microscope (RLM, Axio-scope A1, Carl-Zeis Microscopy GmbH, Munich, Germany) and scanning electron microscope (SEM, JEOL-JSM-5410, Japan), coupled with electron dispersion spectroscopy (EDS, INCA Penta Fe Tx3, Oxford, UK), were used to examine the grain structure and morphology. The Mo, Fe, and oxygen elements were analyzed with EDS. A high-pressure mercury intrusion pore sizer of up to 30,000 psi (Micromertics Instrument Corporation 9320, USA) was also applied for measuring the pore area, pore diameter, bulk density, apparent density, and total porosity in the roasted compacts.

### 2.2. Reduction Procedure

Uniform shape and size compacts from precursors A and B were formed from the pressing of about 2.0 g of a powder sample in a stainless mold, using a hydraulic press. The produced compacts (d = 4 mm, h = 6 mm) were kept dry in a desiccator. The reduction experiments were carried out isothermally at 600–850 °C in purified H_2_ (NETCO, Cairo, Egypt, purity ≥ 99.9%), using a microbalance with a precision of ±0.1 µg (Mettler-Toledo XPR). The O_2_ weight loss that resulted from the reduction reactions was continuously recorded as a function of time. The reduction and gas purification systems were given elsewhere [34]. The preliminary reduction tests indicated that 30 mL/min of the H_2_ gas flow was sufficient to overcome the gas boundary layer effect around the sample. For a given experiment, the tube furnace was heated up to the pre-determined temperature in a continuous flow of purified Ar gas, then kept constant for 10 min. The dry compact, placed inside a platinum perforated basket, was hung from the balance arm with a platinum wire chain. The sample was slowly inserted into the furnace, continued to reach the hot zone, and was left for a while, in order to reach the pre-determined temperature. Under these conditions, the Ar gas was switched off and the H_2_ gas (30 mL/min) was introduced into the reaction tube. The reduction reactions were started and continued until the sample reached a constant weight, showing that no more weight could be recorded. The reduced sample was cooled down to room temperature in a continuous flow of purified Ar gas until room temperature, then dropped in acetone to prevent re-oxidation reactions. The extent of the reduction (*R_t_*) that represents the reduction degree at a given time (*t*) can be calculated from Equation (1), as the following:

*R_t_* (%) = (*W_o_* − *W_f_*)/*W*_0_ × 100 (1)

where *W*_0_ = the original weight of the sample, and *W_t_* = the weight at a given time (*t*).

For the partially reduced samples, the weight of the oxygen content (*W_x_*) at a given reduction extent (*R_x_*) was first calculated, the compact was subjected to the reduction experiment, and the O_2_ weight loss was recorded until it reached the pre-calculated weight loss. Under these conditions, the furnace was switched off and the H_2_ gas was replaced by Ar, and the sample was left to cool down to room temperature, then quickly dropped in acetone. The partially and/or completely reduced samples were analyzed and examined by XRD, (RLM), and SEM-EDS.

## 3. Results and Discussion

### 3.1. Characterization of Materials

**Figure 1:**
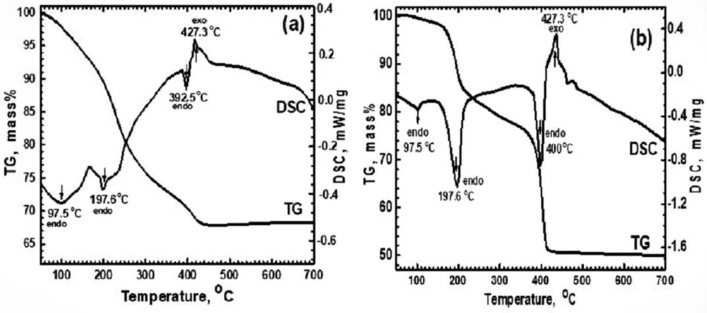
TG-DSC analysis of precursors thermally heated up to 700 °C in Ar gas atmosphere; (**a**) precursor A; and (**b**) precursor B.

The dry powder precipitates from precursors A and B were thermally roasted in an air atmosphere of up to 700 °C using a TG-DSC analyzer, and their thermal behavior are given in Figure 1a,b, respectively. From the TG profile, the measured weight losses were 32.5% and 55.5%, which resulted from the thermal decomposition of the AHM and ferrous oxalate mixtures [35,36,37]. The thermal decomposition of the pure AHM was studied by H. Cavus et al., and they found that it decomposed to ammonium tetra molybdate (ATM) [(NH_4_)_2_Mo_4_O_13_] at 503 K, prior to the formation of MoO_3_ [35]. However, in the present study, different molar ratios of AHM and ferrous oxalate were co-precipitated to produce the precursors, and accordingly, the measured weight loss from the thermal analysis of precursors A and B was dependent on the molar ratio of each component in the precursors. The DSC profiles showed the presence of endothermic peaks at 97.8, 196.9, and 392.5–400 °C, which resulted from the removal of the moisture content, the decomposition of AHM to MoO_3_, and the decomposition of ferrous oxalate to Fe_2_O_3_, respectively. The higher weight loss that was recorded in precursor B in comparison to precursor A resulted from the presence of an excess molar ratio of ferrous oxalate, which was greater than the stoichiometric ratio that produces the iron molybdate. The detection of exo- and endo-thermal peaks in the DSC analysis of both the precursors confirms the presence of more than one thermal decomposition reaction step, as discussed herein after [29].

The thermal decomposition reactions of the AHM and ferrous oxalate are given in Equations (2) and (3), respectively, as follows.

[(NH_4_)_6_ Mo_7_ O_24_.4H_2_O] = 7 MoO_3_ + 6 NH_3_ + 7 H_2_O (2)

2 [FeC_2_O_4_·2H_2_O] + ½ O_2_ = Fe_2_O_3_ + 2 CO + 2 CO_2_ + 4H_2_O (3)

The exothermic peak that was measured at 427.3–430 °C (Figure 1a) resulted from the solid-state reaction between MoO_3_ (Equation (2)) and Fe_2_O_3_ (Equation (3)) to produce the iron molybdate Fe_2_(MoO_4_)_3_, as given in Equation (4):

3MoO_3_ + Fe_2_O_3_ = Fe_2_(MoO_4_)_3_ (4)

The reaction products that were obtained from the thermal treatment of up to 700 °C of precursors A and B, given in Equations (2)–(4), were subjected to an XRD phase analysis that confirmed the above findings. From Figure 1, it can be observed that no more weight loss was recorded at >430 °C, which indicated that the dissociation and solid-state reactions were completed. Accordingly, green compacts were made from precursors A and B and thermally heated at 500 °C for 3 h in an Ar gas atmosphere, and the products were characterized. Table 1 shows the chemical compositions, phases identified, molar ratios, total porosity, and apparent density that were measured in the roasted precursors. The micro- and macro-structures were microscopically examined, and the phases were elementally analyzed by EDS.

It was found that iron molybdate [Fe_2_(MoO_4_)_3_ was the only phase that was identified in precursor A, while in precursor B, α-Fe_2_O_3_ and [Fe_2_(MoO_4_)_3_] were identified. The presence of excess Fe_2_O_3_ in precursor B hindered the grain growth of the iron molybdate, which, in turn, affected the total porosity and apparent density.

**Figure 2:**
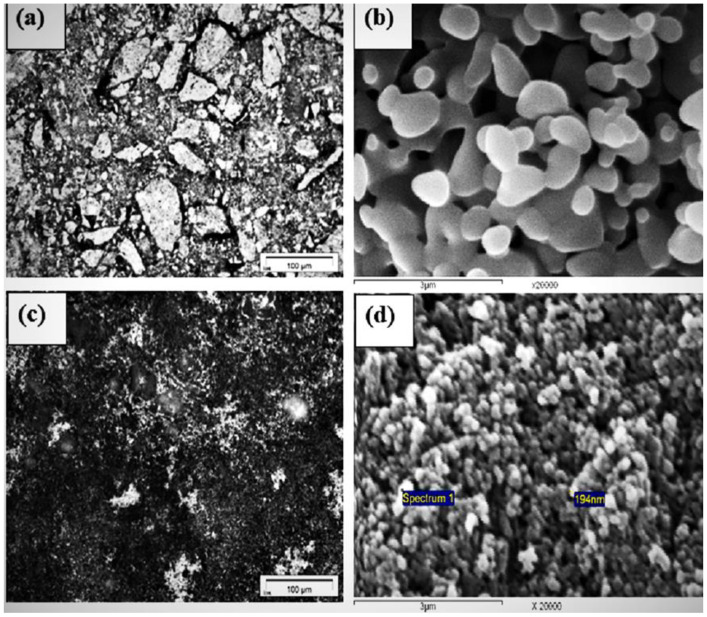
RLM photomicrographs and SEM image for precursor compacts thermally heated up to 500 °C in Ar gas atmosphere; (**a**,**b**) for precursor A; and (**c**,**d**) for precursor B.

Figure 2 shows the RLM photomicrographs and the SEM images for the roasted precursors A and B. The photomicrograph that is given in Figure 2a shows the formation of different grain sizes of the iron molybdate. The larger grains were composed of large numbers of smaller grains, with different shape and sizes that were embedded together in a matrix, which were sintered forming clusters. These grains coalesced with each other, forming aggregates that left large pores. The SEM image that is given in Figure 2b shows the formation of the large sizes of grains, which were bonded together through the neck at the points of collision, forming aggregates. On the other hand, the photomicrographs of precursor B, which are given in Figure 2c, show the presence of relatively large numbers of smaller-size grains, compared to those in precursor A. The smaller grains were mainly composed of hematite, while iron molybdate was in the larger grains. The SEM image for precursor B, which is given in Figure 2d, shows the formation of rounded grains of iron molybdate and hematite, which have an average size of 194 nm. The higher porosity that was measured in precursor A than that in B was due to the presence of larger pores between the agglomerated grains.

### 3.2. Reduction Behavior

#### 3.2.1. Influence of Reduction Temperature

**Figure 3:**
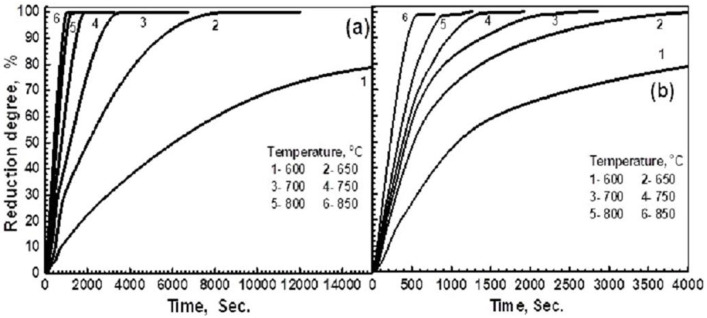
Isothermal reduction behavior of precursors with H_2_ at 600–850 °C; (**a**) precursor A; and (**b**) precursor B.

The compacts from precursors A and B were isothermally reduced at 600–850 °C in the H_2_ gas. The reduction degree at a given time was calculated by applying Equation (1), taking into consideration the maximum O_2_ weight loss that was measured from the reduction reactions represented 100%, since no more weight loss was recorded. Consequently, the relative reduction degrees (%) at a given time were calculated relative to the maximum reducible O_2_ content that was measured in the precursors. The reduction behavior of precursors A and B is shown in Figure 3a,b, respectively. It was found that the reduction at <600 °C was very slow and stopped before completion, depending on the precursor composition. At >850 °C, the reduced products swelled, showing an increase in volume of 175% and 140% for precursors A and B at 900 °C, respectively. For precursor A, composed of 100% iron molybdate, the rate of reduction increased with a rise in temperature and decreased with an increase in the reduction extents. It can be observed that the temperature had a relatively considerable influence on the reduction rate, showing a higher difference up to 750 °C, above which, the difference in the rate was decreased. For a given reduction path at <700 °C, the rate during the early stages was gradually decreased with time, up to certain extent, above which, there was a considerable decrease in the rate until the end of the reduction process. During the early stages, the rate started to decrease from a 30% extent at 750 °C to about 24% at 650 °C, and then to 14% at 600 °C. During the later stages, the rate gradually decreased until the end of the reduction process, showing a slowing in the rate. At ≥750 °C, the reduction of the precursor was fast, up to a certain extent, after which, an abrupt decrease showed a slowing in the rate during the final stages, which decreased with a rise in the temperature. The presence of a slowing in the rate indicated that the reduction could not be continued by a gas–solid reaction, and most probably, the solid-state diffusion contributed to the reduction reactions.

For precursor B, which was composed of 53.32% α-Fe_2_O_3_ and 46.68% Fe_2_(MoO_4_)_3_, the typical reduction curves are shown in Figure 3b. The same behavior can be observed, where the rate of the reduction was increased, increasing the reduction temperature. During the early stages, the reduction path at 600 °C was significantly lower than that at 650 °C, above which, the reduction paths were closer to each other up to 850 °C. During the intermediate stages, the reduction rate was greatly decreased until the end of the reduction. Unlike in precursor A, the reduction at ≤650 °C was completed, and the rate gradually decreased until the end of the reduction reactions.

**Figure 4:**
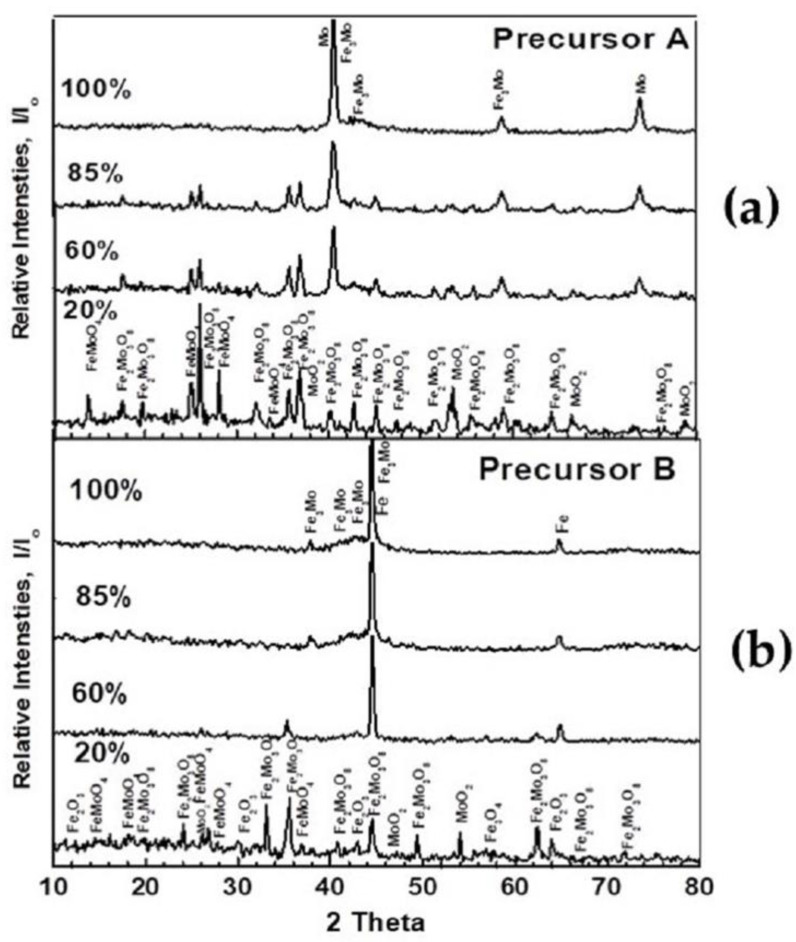
XRD phase analysis of partially and completely reduced precursors at 750 °C with H_2_ at different reduction degrees; (**a**) precursor A; and (**b**) precursor B.

In order to follow up the reduction reactions of the precursors and the formation of the intermediate reaction products, precursors A and B were partially reduced up to 20, 60, and 85% extents. The partially and completely reduced samples were examined by XRD, RLM, and SEM-EDAX. Figure 4a,b show the typical XRD patterns of the partially and completely reduced samples from precursors A and B at 750 °C, respectively. Figure 4a shows that Fe_2_(MoO_4_)_3_ was stepwisely reduced to lower oxides, prior to the formation of the MoFe intermetallic and Mo. Figure 4b shows that, in precursor B, besides the formation of lower oxides from the reduction reactions of Fe_2_(MoO_4_)_3_, Fe_2_O_3_ was also stepwisely reduced to Fe_3_O_4_ and/or wűstite (FeO), and finally to α-Fe. In this precursor, the final reaction products were MoFe, MoFe_3_ intermetallics, and α-Fe.

The different phases that were identified from the reduction reactions of precursors A and B at the 20, 60, 85, and 100% extents are given in Table 2.

From Table 2, it can be seen that Fe_2_(MoO_4_)_3_ was stepwisely reduced to Fe_2_Mo_3_O_8_, FeMoO_4_, and MoO_2_ phases before the final production of the MoFe intermetallics and metallic Mo in precursor A. In precursor B, the stepwise reduction of Fe_2_(MoO_4_)_3_ and Fe_2_O_3_ led to the synthesis of the FeMo and Fe_3_Mo intermetallics and metallic Fe in the final reaction product.

**Figure 5:**
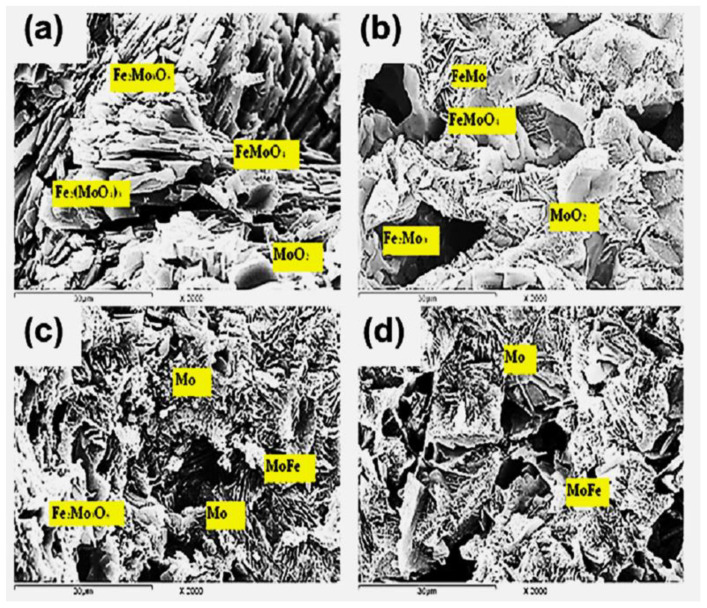
SEM images analysis for precursors (A) reduced at 750 °C with H_2_ up to (**a**) 20%; (**b**) 60%; (**c**) 85%; and (**d**) 100% degrees.

**Figure 6:**
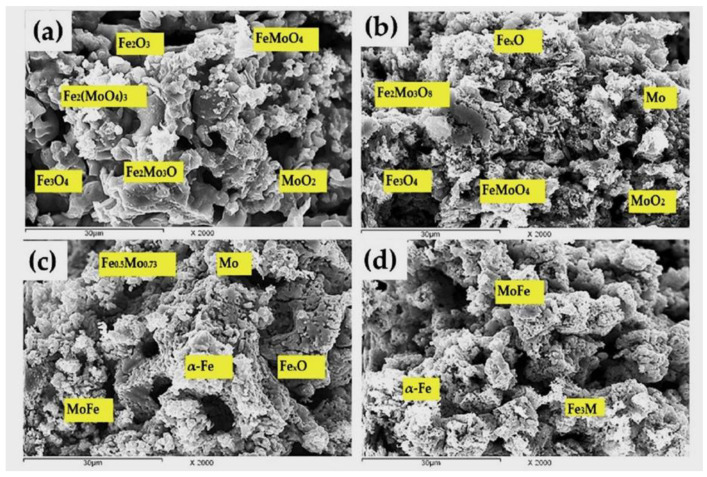
SEM images analysis for precursors (B) reduced at 750 °C with H_2_ up to (**a**) 20%; **(b**) 60%; (**c**) 85%; and (**d**) 100% degrees.

The SEM images for precursors A and B, partially and completely reduced at 750 °C (up to 20%, 60%, 85%, and 100% extents), are given in Figure 5 and Figure 6, respectively. EDS was applied to analyze the Mo, Fe, and oxygen in the different phases. Figure 5a shows the structure of the 20% reduced compact, in which Fe_2_(MoO_4_)_3_ is reduced to [Fe_2_Mo_3_O_8_] in a needle shape and has a parallel orientation alongside the presence of the irregular grains of MoO_2_. With an increase in the reduction extent of up to 60% (Figure 5b), FeMoO_4_ is further reduced to MoO_2_ and FeMoO_4_, while Fe_2_Mo_3_O_8_ is still also identified. In the 85% reduced sample (Figure 5c), Fe_2_Mo_3_O_8_, MoFe, and Mo are present. In the completely reduced precursor (Figure 5d), MoFe intermetallics are the predominant phase that is identified in the form of the whiskers together, with small grains of Mo metal. Unlike the grain structure that was developed in precursor A, the grain structure that was formed in precursor B differs, due the presence of an excess molar ratio of iron oxides. The grain structure of the 20% reduced sample, which is given in Figure 6a, shows the presence of Fe_2_O_3_ and Fe_3_O_4_ in smaller grain sizes, alongside the lower oxides of the iron molybdate in different grain structures. The needles structure of Fe_2_Mo_3_O_8_ is still present. The FeMoO_4_ phase is present in dense grains, while MoO_2_ is formed in the matrix as smaller grains. The structure that developed in the 60% reduced sample (Figure 6b) shows the presence of magnetite and wüstite from the reduction of Fe_2_O_3_. In this precursor, Fe_2_Mo_3_O_8_, FeMoO_4_, and MoO_2_ are also present. In the 85% reduced sample, MoFe intermetallics are clearly identified in the dense grains that are connected to each other, forming clusters, while the FeMoO_4_ phase is still present, together with the Mo metal, as given in Figure 6c. In the completely reduced precursor (Figure 6d), MoFe_3_, MoFe intermetallics, and metallic iron are present.

#### 3.2.2. Influence of Precursor Composition

**Figure 7:**
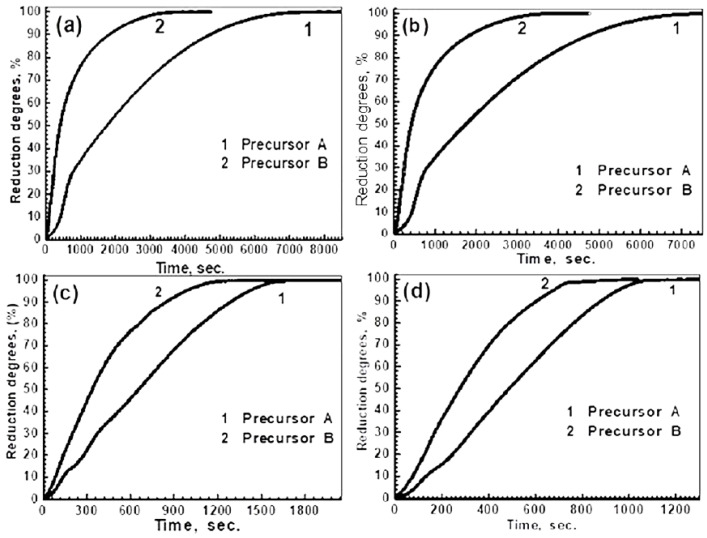
Influence of precursor composition on the isothermal reduction behavior with H_2_ at (**a**) 650 °C; (**b**) 700 °C; (**c**) 750 °C; and (**d**) 800 °C.

The isothermal reduction behavior of precursors A and B with H_2_, at 650, 700, 750, and 800 °C, are given in Figure 7a–d, respectively. It can be seen that the rate of reduction for precursor B is higher than that for precursor A, at all temperatures, and proceeds smoothly, in which the rate gradually decreases with an increase in the reduction extent until the end of reduction process. On the other hand, the reduction of precursor A shows that the rate of reduction is lower during the early stages, depending on the reduction temperature, then increases up to about a 20% extent, above which, the rate gradually decreases to about a 95% extent. During the later stages, a slowing in the rate of reduction is clearly identified, with special emphasis on precursor A rather than B. This is mainly due to the presence of excess Fe_2_O_3_ than the stoichiometric ratio which is required to produce iron molybdate.

### 3.3. Chemistry of Intermetallics Formation

The reduction reactions of the Fe_2_(MoO_4_)_3_ and Fe_2_O_3_ phases in the precursors with H_2_ were found to proceed through the formation of intermediate reaction products, prior to the synthesis of the MoFe and MoFe_3_ intermetallic alloys, as previously given in Table 2. Based on the intermediate reaction phases that were identified at the different reduction extents, the chemical reaction equations that describe the reduction reactions of the precursors can be formulated as follows:

1. The chemical reaction equations of precursor A, [100% Fe_2_(MoO_4_)_3_]

2 [Fe_2_(MoO_4_)_3_] + 6 H_2_ = 2 FeMoO_4_ + Fe_2_Mo_3_O_8_ + MoO_2_ + 6 H_2_O (at 20% reduction degree) (5)

2 FeMoO_4_ + 8 H_2_ = 2 FeMo + 8 H_2_O (at 60% reduction degree) (6)

Fe_2_Mo_3_O_8_ + 8 H_2_ = 2FeMo + Mo + 8 H_2_O (at 85% reduction degree) MoO_2_ + 2 H_2_ = Mo + 2 H_2_O (7)

2 [Fe_2_(MoO_4_)_3_] + 24 H_2_ = 4FeMo + 2 Mo + 24 H_2_O (at 100% reduction degree) (8)

2. The chemical reaction equations of precursor (B), [46.68% Fe_3_(MoO4)_3_ + 53.32% Fe_2_O_3_];

2 [Fe_2_(MoO_4_)_3_] + 24 H_2_ = 4 FeMo + 2 Mo + 24 H_2_O (reduction of iron molybdate) (9)

4 Fe_2_O_3_ + 12 H_2_ = 8 Fe + 12 H_2_O (reduction of iron oxide) (10)

8 Fe + 2Mo = 2 Fe_3_Mo + 2Fe (formation of intermetallic) (11)

2 [Fe_2_(MoO_4_)_3_] + 4Fe_2_O_3_ + 36H_2_ = 4FeMo + 2Fe_3_Mo + 2Fe+ 36 H_2_O (at a 100% reduction degree) (12)

From the chemical reaction equations, depending on the precursor composition, it can be reported that the MoFe intermetallics and Mo are obtained from the reduction of precursor A, while MoFe, the MoFe_3_ intermetallics, and Fe are produced from the reduction of precursor B. Such a conclusion agrees with the XRD phase analysis that is given in Table 2 and the microstructure changes that are given in Figure 5 and Figure 6.

### 3.4. Reduction Kinetics and Mechanisms

Thermodynamic analysis is an important issue in the synthesis and characterization of intermetallic alloys from their original material, via gas–solid reaction processes. The mechanism of the reduction reaction can be predicted from both the apparent activation energy (*E_a_*) values, which are calculated from Arrhenius plots, and also from the application of the mathematical formulations that are derived from the gas–solid model.

The apparent activation energy values (*E_a_*) can be calculated from the application of the Arrhenius equation [38];

*K_r_* = *K_o_*. *e*^−*Ea/RT*^ (13)

**Figure 8:**
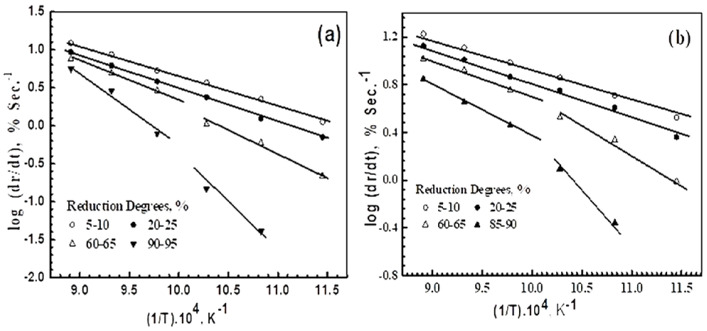
Arrhenius plots for the reduction of precursors with H_2_ at 600–850 °C: (**a**) precursor A; and (**b**) precursor B.

Where *K_r_* is the reduction rate constant, *K_o_* is the frequency factor, R is the gas constant, and *T* is the absolute temperature. Figure 8a,b represent the Arrhenius plots, in which the (*log dr/dt*) values that were calculated at the different reduction extents are plotted against the corresponding (1*/T*) for precursors (A) and (B), respectively. The *E_a_* values that were computed during the early, intermediate, and later stages of the reduction, together with the proposed reduction mechanism, are given in Table 3.

The calculated *E_a_* values can be used to predict the reduction mechanism. For precursor A, the reduction during the early stages (up to 10%) is controlled by the combined effects of the gas diffusion and interfacial chemical reaction mechanism. With an increase in the reduction extent, the contribution of the interfacial chemical reaction increases until it becomes the rate-controlling mechanism, up to about an 85% extent, which is dependent on the applied temperature. During the later stages (≥90% extent), the reduction of precursor A at ≥750 °C is still controlled by the interfacial chemical reaction. At ≤700 °C, the reduction reaction is controlled by the solid-state diffusion mechanism, resulting in the slowing of the rate until the end of the reduction reaction.

On the other hand, the reduction of precursor B is controlled by the gaseous diffusion mechanism up to a 10% extent, after which, the contribution of the interfacial chemical reaction increases until it becomes the rate-determining mechanism, up to about a 90% extent. During the later stages, the interfacial chemical reaction is the rate-controlling mechanism at ≥750 °C. At ≤ 700 °C, the solid-state diffusion seems to be the rate-controlling mechanism until the end of the reduction process.

In another way to confirm our findings regarding the mechanisms of reduction, the gas–solid reaction model [11,32,33,39] was performed. The simplified formulations of the gas diffusion, interfacial chemical reaction, and mixed control mechanism that were derived from the gas–solid reaction model are:

*P_Fg_* (*X*) = [*X* + (1 − *X*) *ln* (1 − *X*)] = *K*_1_*t* (gaseous diffusion control) (14)

t* = [ (1 − (1 − *X*) ^1/*Fg*^] = *K*_2_*t* (interfacial chemical reaction control) (15)

t* = [*X* + (1 − *X*) *ln* (1 − *X*)] + [ (1 − (1 − *X*) 1/*Fg*] = *K*_3_*t* (mixed control reaction) (16)

where *K*_1_*, K*_2_*,* and *K*_3_ are constants (sec^−1^); *X* is the fractional reduction degree (*Rt/*100) calculated at a given time (*t*), as defined in Equation (1); t* is the dimensionless time; and *Fg* is the shape factor (1 and 2 for grains and compacts, respectively).

**Figure 9:**
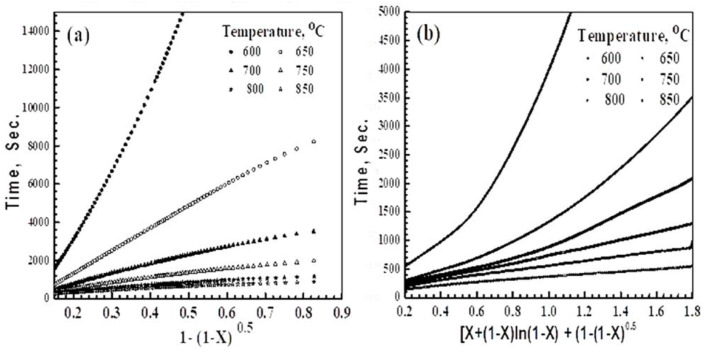
Application of mathematical equations for the reduction of precursors at 600–850 °C in H_2_: (**a**) precursor A; and (**b**) precursor B.

The mathematical formulations that are given in Equations (14)–(16) were tested against the experimental reduction reaction results of precursors A and B. For precursor A, the testing of the three mathematical equations resulted in only straight lines upon applying Equation (15), as shown in Figure 9a. These straight lines are valid from 15%, up to certain extent, depending on the applied temperature. At ≥750 °C, the straight lines are valid up until the later stages of the reduction, which indicates that the reduction reaction is controlled by the interfacial chemical reactions mechanism. At <700 °C, the straight lines are deviated at the different reduction extents (Figure 9a). This indicates that the reduction is controlled by the interfacial chemical reaction mechanism, up to certain extent, beyond which, the solid-state diffusion contributes with the interfacial chemical reaction and becomes the rate-determining mechanism.

Figure 9b shows the application of the mixed control reaction formulation (Equation (16)) against the reduction results of precursor B. The presence of two sets of lines can be observed, in which straight lines are obtained at ≥750 °C, beyond which, the straight lines are deviated at different extents, depending on the applied temperatures. This indicates that the reduction is controlled by the combined effects of gas diffusion and the interfacial chemical reaction mechanism (mixed control mechanism). At higher temperatures, the contribution of the interfacial chemical reaction in the mixed mechanism increased with an increase in the reduction extents, and decreased with a rise in temperature. At ≤ 700 °C, the reduction is controlled by the mixed reaction mechanism, up to a certain extent, and then by the interfacial chemical reaction mechanism until the end of the reduction process.

## 4. Conclusions

The gas–solid reaction technique is successfully applied to produce nano-structured MoFe and/or MoFe_3_ intermetallics from the reduction of precursors that contained 72/28 and 30/70% molar ratios, named as precursors A and B, respectively. Both precursors were prepared from the co-precipitation of AHM and ferrous oxalate. Their roasted compacts were isothermally reduced in H_2_ at 600–850 °C. The reduction of precursor A showed that [Fe_2_(MoO_4_)_3_] was reduced to lower intermediate oxides, prior to the production of the MoFe intermetallic and Mo. On the other hand, MoFe and MoFe_3_ intermetallics, together with metallic Fe, were obtained from the reduction reactions of precursor B. The rate of reduction of precursor B was much higher than that of precursor A, at all temperatures, due to the presence of an excess molar ratio of Fe_2_O_3_, which was greater than the stoichiometric molar ratio that produces [Fe_2_(MoO_4_)_3_]. Depending on the intermediate reaction products, which were identified by XRD and examined by SEM-EDS, the chemical reaction equations that were given aimed at following up the synthesis of the intermetallic alloys (MoFe and MoFe_3_).

The mechanisms of the reduction reactions of precursors A and B during the early, intermediate, and later stages were elucidated from the correlations between the computed activation energy (*E_a_*) values and the applications of the mathematical formulations that were derived from the gas–solid reaction model. The reduction mechanism was greatly dependent on the precursor composition and reduction temperature. It was concluded that the reduction reaction mechanism of precursor A was controlled by the combined effect of a gas diffusion and an interfacial chemical reaction mechanism, up to about a 15% extent, above which, the reduction reaction was controlled by the interfacial chemical reaction, until about a 85% extent. With progress in the reduction process, the solid state-diffusion contributed, with the interfacial chemical reaction, as the rate-controlling mechanism. During the later stages, the contribution of the solid-state diffusion increased and it became the rate-controlling mechanism. The reduction of precursor B was controlled by gas diffusion during its early stages, followed by higher contribution of the interfacial chemical reaction until the later reduction stages. During the final stages, the solid-state diffusion slightly contributed with the interfacial chemical reaction.

From this study, it can be concluded that the gas–solid reaction technique can be successfully applied to produce nano-structured and nano-sized intermetallic alloys. A reduction with H_2_ gas is considered to be an environmentally friendly approach and can be extended to the synthesis of binary and tertiary metal alloys.

## Figures and Tables

**Table 1:** Characteristics and phases identified in precursors roasted at 500 °C for 3 h.

Precursor	Composition		Phases Identified	Molar Ratio (%)	Total Porosity (%)	Apparent Density (g/mL)
	Mo %	Fe %				
(A)	72	28	Fe_2_(MoO_4_)_3_	100	59.87	6.43
(B)	30	70	Fe_2_(MoO_4_)_3_ α-Fe_2_O_3_	46.68 53.32	48.39	5.35

**Table 2:** XRD phase analysis of partially and completely reduced samples from precursors A and B at 750 °C.

Precursor Composition	Phases Identified at Different Reduction Extents			
	20%	60%	85%	100%
Precursor A 100% Fe_2_(MoO_4_)_3_	Fe_2_(MoO_4_)_3_, Fe_2_Mo_3_O_8_ FeMoO_4_, MoO_2_	Fe_2_Mo_3_O_8,_ FeMoO_4_, FeMo, MoO_2_	Fe_2_Mo_3_O_8_, MoFe, Mo	MoFe, Mo
Precursor B 46.68% Fe_2_(MoO_4_)_3_ +53.32% Fe_2_O_3_	Fe_2_(MoO_4_)_3_, Fe_2_Mo_3_O_8_ FeMoO_4_, MoO_2_, Fe_2_O_3_, Fe_3_O_4_	Fe_2_Mo_3_O_8,_ FeMoO_4_ Mo, Fe_3_O_4,_ Fe_x_O, MoO_2_	Fe_0_._5_Mo_0_._73,_ MoFe, Mo, Fe_x_O, α-Fe	MoFe, Fe_3_Mo, α-Fe

**Table 3:** *E_a_* values computed for the reduction of precursors at 600–850 °C and the proposed corresponding reaction mechanism.

Reduction Degree, %		*Ea*, kJ mole^−1^		Rate Controlling Mechanism According to the Reference Values of Activation Energy, [29]	
		Precursor (A)	Precursor (B)		
5–10%		25.63	15.87	*E_a_*, (kJ mole^−1^)	Controlling mechanism
20–25%		48.09	32.31	8–16	Gas diffusion
60–70%		65.23	48.98	29–42	Gas diffusion and interfacial chemical reaction
90–95%	at ≥750 °C	85.32	65.85	60–70	Interfacial chemical reaction
	at ≤700 °C	125.79	94.85	>90	Solid-state diffusion

## Data Availability

Not applicable.
